# Supplementary material for: Molecular basis of PIP2-dependent regulation of the Ca2+-activated chloride channel TMEM16A
Source: Nat Commun. 2019 Aug 21;10:3769. doi: 10.1038/s41467-019-11784-8 (PMC6704070; doi:10.1038/s41467-019-11784-8)
Supplement: Supplementary file 1 — Supplementary Information [file 41467_2019_11784_MOESM1_ESM.pdf]

Molecular basis of PIP$_2$-dependent regulation of the Ca^{2+}-activated chloride channel TMEM16A

Son C. Le, Zhiguang Jia, Jianhan Chen, Huanghe Yang*

This PDF file includes:

Supplementary Figs. 1-14
Supplementary Table 1. Primers for QuikChange mutagenesis

Other Supplementary Materials for this manuscript include the following:

Supplementary Movies 1 and 2.
Source Data File for Figs. 1b-d,f,g, 2, 3a,c, 5d-g, 6a-d,f, and Supplementary Figs. 1, 2, 3, 4, 6, 7, 8, 9a, 11, 12, 13, 14.

*Correspondence to:
Huanghe Yang
Email: huanghe.yang@duke.edu
Telephone: 919-684-1406

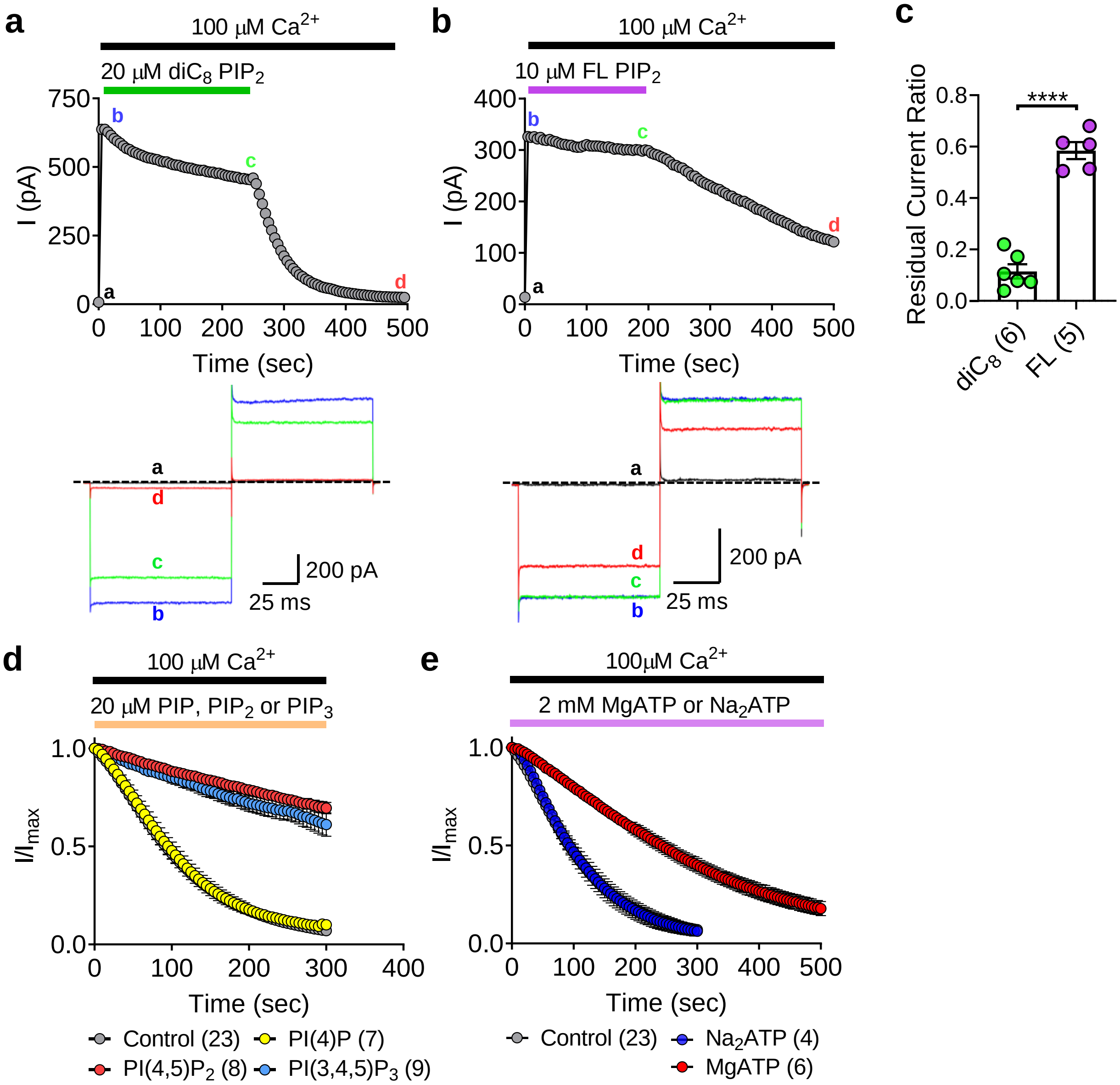

Supplementary Fig. 1. Functional characterizations of diC8 PIP2 and full-length PIP2 on TMEM16A. a,**b**, Representative recordings showing the effects of 20 µM diC8 PIP2 (**a**) and 10 µM FL PIP2 (**b**) on TMEM16A desensitization. Current traces at different time points are shown as insets on the right. **c**, Residual ratio of TMEM16A current after withdrawing diC8 PIP2 and FL PIP2 (d, at ~500 s) to the initial current (c). Two-tailed unpaired Student's *t*-test: p-value is <0.0001. **d**, diC8 PI(4,5)P2 and diC8 PI(3,4,5)P3, but not diC8 PI(4)P, attenuated TMEM16A channel desensitization under saturating 100 µM Ca^{2+}. **e**, 2 mM MgATP, but not Na2ATP, slowed down TMEM16A desensitization likely by promoting phosphoinositide synthesis. Numbers in parentheses denote the number of individual recordings. Data are mean ± s.e.m. Source data are provided as a Source Data file.

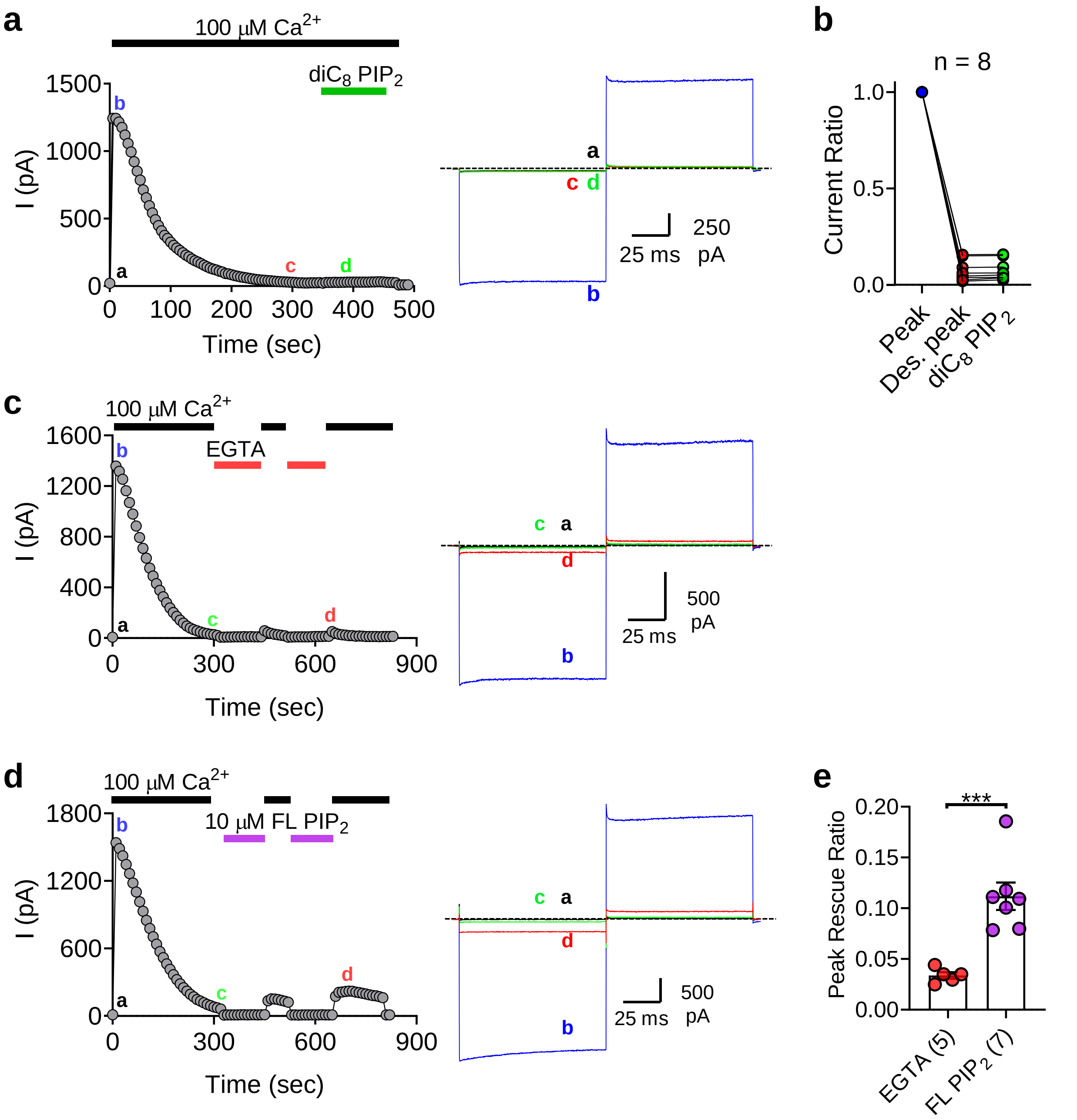

Supplementary Fig. 2. FL PIP$_2$ partially rescues TMEM16A after desensitization. **a**, 20 μM short-chain diC$_8$ PIP$_2$ fails to rescue the desensitized TMEM16A channels under saturating 100 μM intracellular Ca^{2+}. Representative current traces at different time points are shown on the right. **b**, Normalized current amplitudes from recordings shown in **a**. Peak, normalized maximal current; Des. peak, current after 5 minutes of Ca^{2+} application (normalized to maximal peak); diC$_8$ PIP$_2$, current after 20 μM diC$_8$ PIP$_2$ application in addition to 100 μM Ca^{2+} (normalized to peak). **c**,**d**, Representative recordings showing application of 10 μM full-length PIP$_2$ (**d**), but not EGTA (5 mM) control solution (**c**), partially rescued TMEM16A channel activity following desensitization. Notice the more sustained channel opening following FL PIP$_2$ application. Representative current traces at different time points are shown on the right. Voltage step protocol of -80 mV and +80 mV lasting 100 ms each with the membrane held at 0 mV was used. **e**, The extent of channel rescue

by EGTA and FL PIP_2 (purple) as quantified by the second peak rescue (point d) versus the original peak current (point a). Two-tailed unpaired Student's *t*-test: p-value is <0.0001. Data are mean ± s.e.m. Source data are provided as a Source Data file.

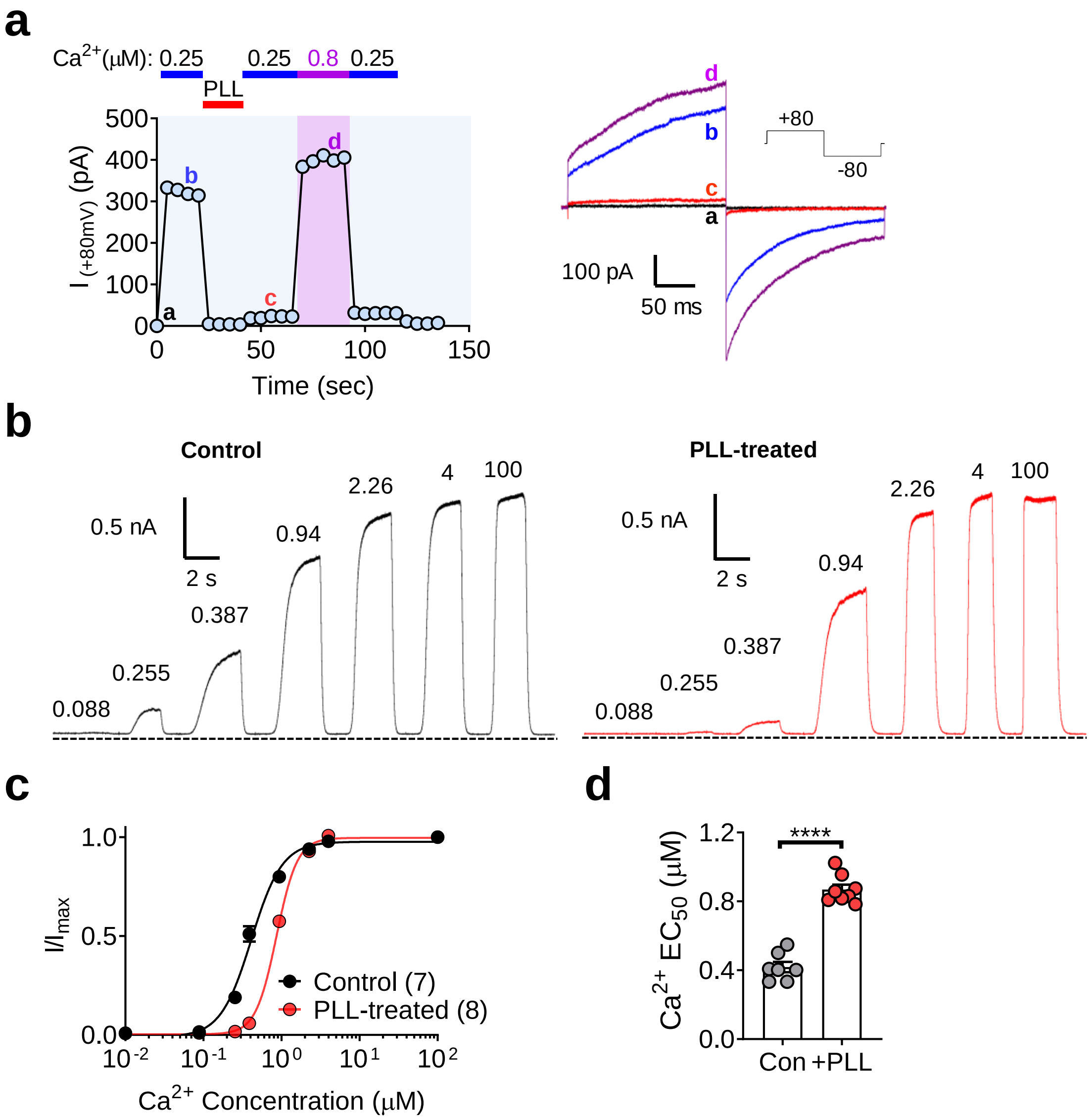

Supplementary Fig. 3. PIP_2 depletion reduces TMEM16A's Ca^{2+} sensitivity. a, Higher Ca^{2+} (0.8 μM) relieved PIP_2 depletion-induced desensitization of TMEM16A under 0.25 μM Ca^{2+} as measured by the steady currents at +80 mV. Representative raw current traces from different time points in **a** are shown on the right (n = 6 independent recordings). **b**, Representative recordings showing Ca^{2+} concentration-dependent activation of TMEM16A in control condition (Control) and after PLL application (PLL-treated). Membrane potential was held at +60 mV. Numbers above each Ca^{2+}-elicited outward current denote intracellular Ca^{2+} concentrations (in μM). **c**,**d**, Ca^{2+} dose-response curves (**c**) of TMEM16A in control and PLL-treated conditions and their half-activation concentrations of Ca^{2+} (EC_{50}) (**d**). Two-tailed unpaired Student's *t*-test: p-value is <0.0001. Numbers in parentheses denote the number of individual recordings. Data are mean ± s.e.m. Source data are provided as a Source Data file.

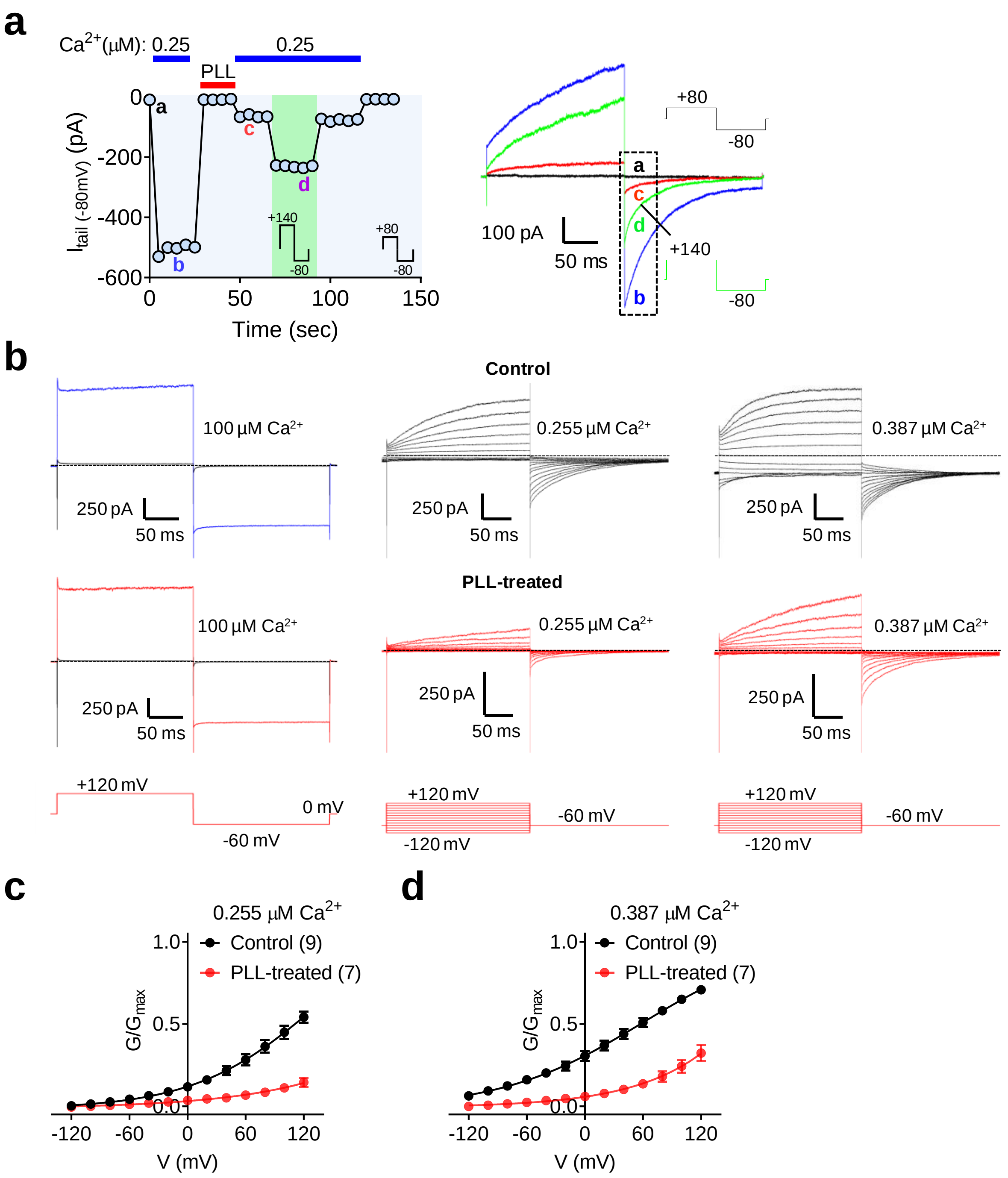

Supplementary Fig. 4. PIP_2 depletion reduces TMEM16A's voltage-sensitivity. a, Increase in membrane depolarization (+140 mV from +80 mV) also partially relieved PIP_2 depletion-induced desensitization of TMEM16A under 0.25 µM Ca^{2+}. Tail currents (I_{tail}) measured at -80 mV (dotted box) were used for analysis to avoid the changes in the driving force resulting from the changes in the depolarization steps (from +80 mV to +140 mV). Representative raw current traces from different time points are shown on the right (n = 8 independent recordings). Voltage step protocols used at different time points are shown in insets. **b**, Representative current-voltage relationship (I-V) recordings of TMEM16A in control condition (Control) and in PIP_2-depleted condition (PLL-

treated). In control condition, the membrane patch was first exposed to 100 μM Ca^{2+} to obtain maximal channel activation with a voltage step protocol in which the membrane was depolarized to +120 mV and repolarized to -60 mV. Using the same patch, 0.255 μM or 0.387 μM Ca^{2+} was applied to elicit voltage-dependent channel activation. In PLL-treated condition, the membrane patch was first exposed to 100 μg/ml PLL for 15-17 seconds before applying 100 μM Ca^{2+} to obtain maximal channel activation, which was followed by voltage-dependent activation by 0.255 μM or 0.387 μM Ca^{2+}. **c**,**d**, Conductance-voltage (G-V) relationship curves in control and PLL-treated conditions. Tail currents measured at the -60 mV repolarization steps following each test voltage were normalized to the steady-state tail current measured under 100 μM Ca^{2+} and were fit with a Boltzmann equation (see Methods for details). Numbers in parentheses denote the number of individual recordings. Data are mean ± s.e.m. Source data are provided as a Source Data file.

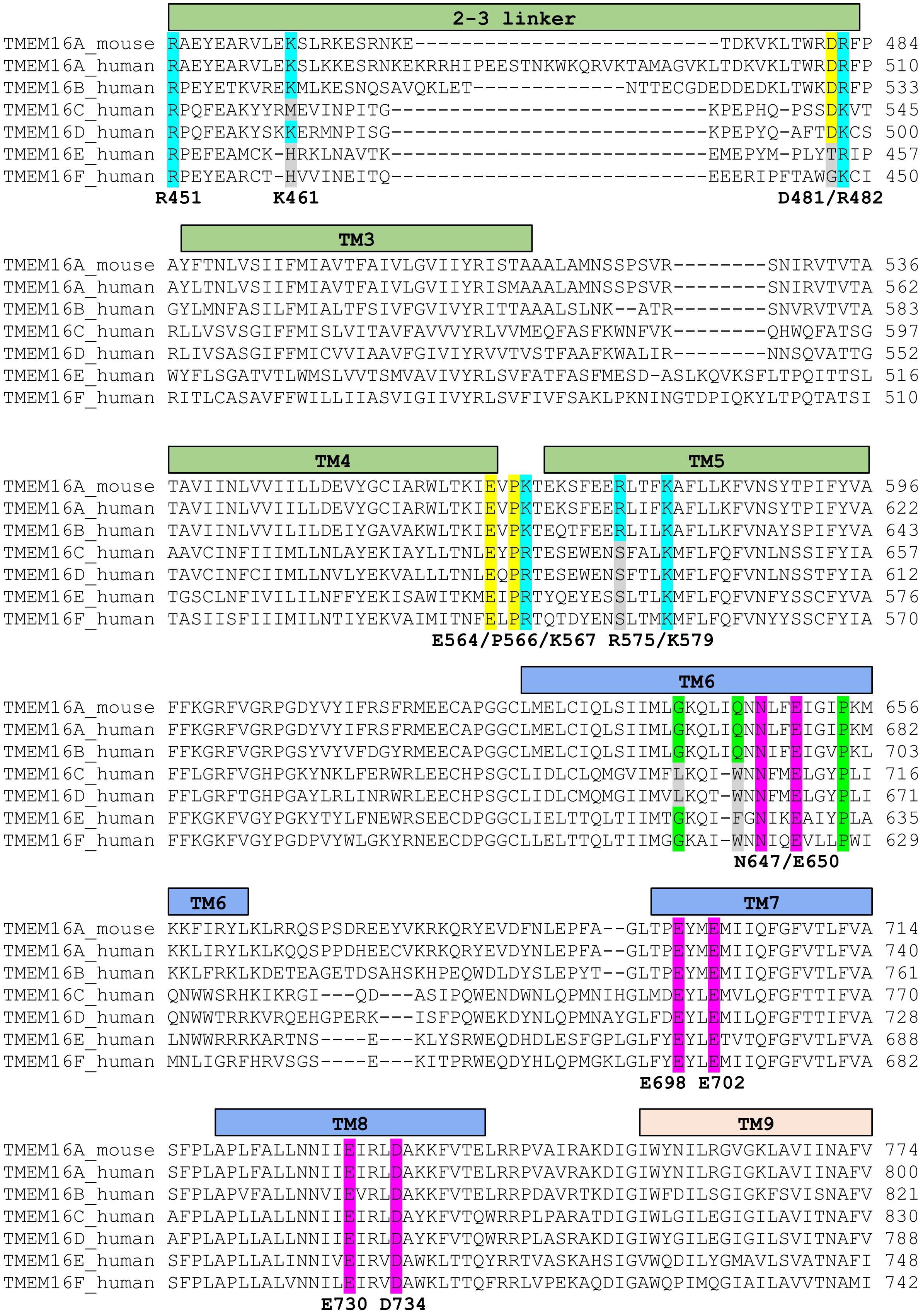
2-3 linker
TMEM16A_mouse RAEYEARVLEKSLRKESRNKE---------------------------TDKVKLTWRDRFP 484
TMEM16A_human RAEYEARVLEKSLKKESRNKEKRRHIPEESTNKWKQRVKTAMAGVKLTDKVKLTWRDRFP 510
TMEM16B_human RPEYETKVREKMLKESNQSAVQKLET-------------NTTECGDEDDEDKLTWKDRFP 533
TMEM16C_human RPQFEAKYYRMEVINPITG----------------------------KPEPHQ-PSSDKVT 545
TMEM16D_human RPQFEAKYSKKERMNPISG----------------------------KPEPYQ-AFTDKCS 500
TMEM16E_human RPEFEAMCK-HRKLNAVTK----------------------------EMEPYM-PLYTRIP 457
TMEM16F_human RPEYEARCT-HVVINEITQ----------------------------EEERIPFTAWGKCI 450
R451 K461 D481/R482
TM3
TMEM16A_mouse AYFTNLVSIIFMIAVTFAIVLGVIIYRISTAAALAMNSSPSVR--------SNIRVTVTA 536
TMEM16A_human AYLTNLVSIIFMIAVTFAIVLGVIIYRISMAAALAMNSSPSVR--------SNIRVTVTA 562
TMEM16B_human GYLMNFASILFMIALTFSIVFGVIVYRITTAAALSLNK--ATR--------SNVRVTVTA 583
TMEM16C_human RLLVSVSGIFFMISLVITAVFAVVVYRLVVMEQFASFKWNFVK--------QHWQFATSG 597
TMEM16D_human RLIVSASGIFFMICVVIAAVFGIVIYRVVTVSTFAAFKWALIR--------NNSQVATTG 552
TMEM16E_human WYFLSGATVTLWMSLVVTSMVAVIVYRLSVFATFASFMESD-ASLKQVKSFLTPQITTSL 516
TMEM16F_human RITLCASAVFFWILLIIASVIGIIVYRLSVFIVFSAKLPKNINGTDPIQKYLTPQTATSI 510
TM4
TM5
TMEM16A_mouse TAVIINLVVIILLDEVYGCIARWLTKIEVPKTEKSFEERLTFKAFLLKFVNSYTPIFYVA 596
TMEM16A_human TAVIINLVVIILLDEVYGCIARWLTKIEVPKTEKSFEERLIFKAFLLKFVNSYTPIFYVA 622
TMEM16B_human TAVIINLVVILILDEIYGAVAKWLTKIEVPKTEQTFEERLILKAFLLKFVNAYSPIFYVA 643
TMEM16C_human AAVCINFIIIMLLNLAYEKIAYLLTNLEYPRTESEWENSFALKMFLFQFVNLNSSIFYIA 657
TMEM16D_human TAVCINFCIIMLLNVLYEKVALLLTNLEQPRTESEWENSFTLKMFLFQFVNLNSSTFYIA 612
TMEM16E_human TGSCLNFIVILILNFFYEKISAWITKMEIPRTYQEYESSLTLKMFLFQFVNFYSSCFYVA 576
TMEM16F_human TASIISFIIIMILNTIYEKVAIMITNFELPRTQTDYENSLTMKMFLFQFVNYYSSCFYIA 570
E564/P566/K567 R575/K579
TM6
TMEM16A_mouse FFKGRFVGRPGDYVYIFRSFRMEECAPGGCLMELCIQLSIIMLGKQLIQNNLFEIGIPKM 656
TMEM16A_human FFKGRFVGRPGDYVYIFRSFRMEECAPGGCLMELCIQLSIIMLGKQLIQNNLFEIGIPKM 682
TMEM16B_human FFKGRFVGRPGSYVYVFDGYRMEECAPGGCLMELCIQLSIIMLGKQLIQNNIFEIGVPKL 703
TMEM16C_human FFLGRFVGHPGKYNKLFERWRLEECHPSGCLIDLCLQMGVIMFLKQI-WNNFMELGYPLI 716
TMEM16D_human FFLGRFTGHPGAYLRLINRWRLEECHPSGCLIDLCMQMGIIMVLKQT-WNNFMELGYPLI 671
TMEM16E_human FFKGKFVGYPGKYTYLFNEWRSEECDPGGCLIELTTQLTIIMTGKQI-FGNIKEAIYPLA 635
TMEM16F_human FFKGKFVGYPGDPVYWLGKYRNEECDPGGCLLELTTQLTIIMGGKAI-WNNIQEVLLPWI 629
N647/E650
TM6
TM7
TMEM16A_mouse KKFIRYLKLRRQSPSDREEYVKRKQRYEVDFNLEPFA--GLTPEYMEMIIQFGFVTLFVA 714
TMEM16A_human KKLIRYLKLKQQSPPDHEECVKRKQRYEVDYNLEPFA--GLTPEYMEMIIQFGFVTLFVA 740
TMEM16B_human KKLFRKLKDETEAGETDSAHSKHPEQWDLDYSLEPYT--GLTPEYMEMIIQFGFVTLFVA 761
TMEM16C_human QNWWSRHKIKRGI---QD---ASIPQWENDWNLQPMNIHGLMDEYLEMVLQFGFTTIFVA 770
TMEM16D_human QNWWTRRKVRQEHGPERK---ISFPQWEKDYNLQPMNAYGLFDEYLEMILQFGFTTIFVA 728
TMEM16E_human LNWWRRRKARTNS----E---KLYSRWEQDHDLESFGPLGLFYEYLETVTQFGFVTLFVA 688
TMEM16F_human MNLIGRFHRVSGS----E---KITPRWEQDYHLQPMGKLGLFYEYLEMIIQFGFVTLFVA 682
E698 E702
TM8
TM9
TMEM16A_mouse SFPLAPLFALLNNIIEIRLDAKKFVTELRRPVAIRAKDIGIWYNILRGVGKLAVIINAFV 774
TMEM16A_human SFPLAPLFALLNNIIEIRLDAKKFVTELRRPVAVRAKDIGIWYNILRGIGKLAVIINAFV 800
TMEM16B_human SFPLAPVFALLNNVIEVRLDAKKFVTELRRPDAVRTKDIGIWFDILSGIGKFSVISNAFV 821
TMEM16C_human AFPLAPLLALLNNIIEIRLDAYKFVTQWRRPLPARATDIGIWLGILEGIGILAVITNAFV 830
TMEM16D_human AFPLAPLLALLNNIIEIRLDAYKFVTQWRRPLASRAKDIGIWYGILEGIGILSVITNAFV 788
TMEM16E_human SFPLAPLLALINNIVEIRVDAWKLTTQYRRTVASKAHSIGVWQDILYGMAVLSVATNAFI 748
TMEM16F_human SFPLAPLLALVNNILEIRVDAWKLTTQFRRLVPEKAQDIGAWQPIMQGIAILAVVTNAMI 742
E730 D734

Supplementary Fig. 5. Protein sequence alignment of TMEM16 proteins. Colored labeled bars indicate transmembrane regions and linkers. Highlighted in cyan are putative PIP_2 binding residues. Highlighted in yellow and green are critical non-basic residues D481, E564, P566 from the PIP_2 module and G640, Q645, and P654 from the Ca^{2+} module. Highlighted in magenta are the highly conserved Ca^{2+}-binding residues from TMs 6, 7, and 8.

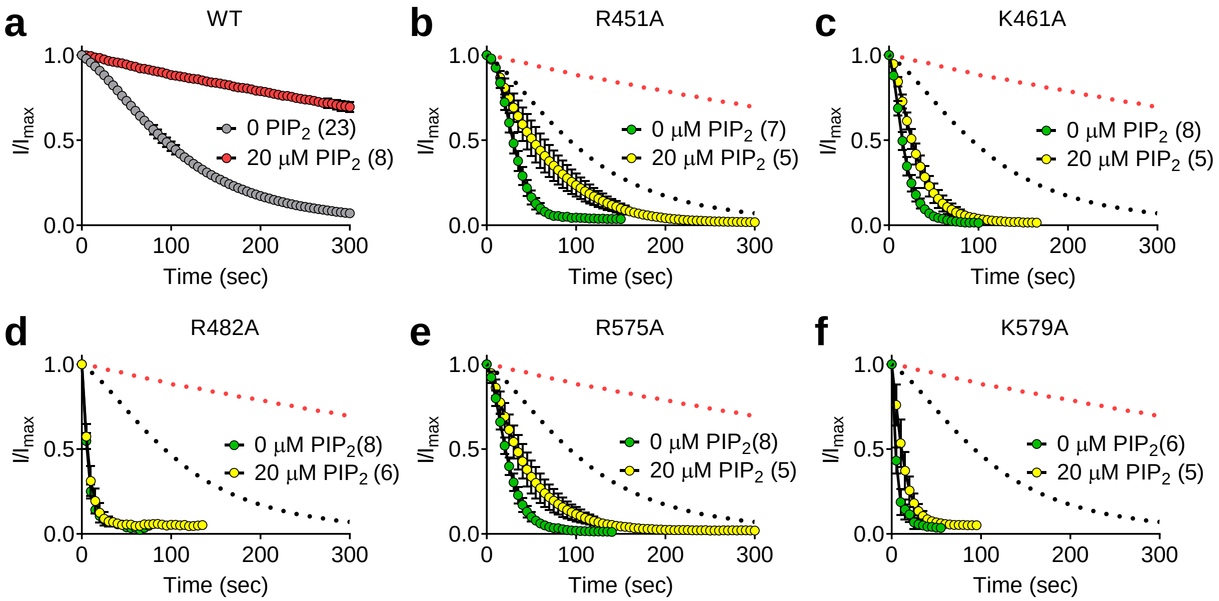

Supplementary Fig. 6. Characterizations of mutations of the putative PIP_2 binding residues under saturating Ca^{2+}. a-f, Average normalized currents showing the effects of diC_8 PIP_2 on channel desensitization of TMEM16A WT (**a**, control in gray and in 20 μM diC_8 PIP_2 in red), R451A (**b**), K461A (**c**), R482A (**d**), R575A (**e**), and K579A (**f**) in the absence of diC_8 PIP_2 (green data points) or in the presence of 20 μM diC_8 PIP_2 (yellow data points). WT TMEM16A desensitization (black dotted line) and its response to 20 μM diC_8 PIP_2 (red dotted line) are shown as controls. Numbers in parentheses denote the number of individual recordings. Data are mean ± s.e.m. Source data are provided as a Source Data file.

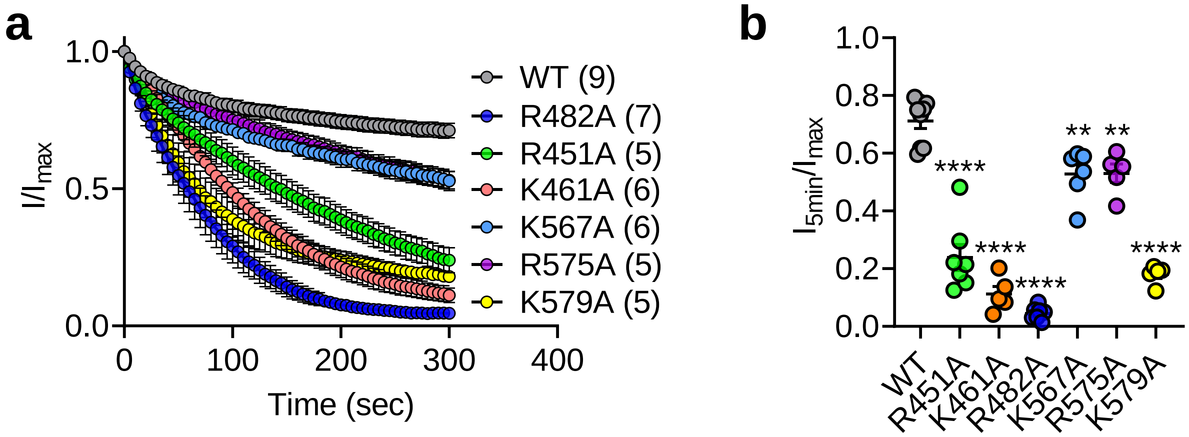

Supplementary Fig. 7. Characterizations of mutations of putative PIP_2 binding residues in sub-micromolar Ca^{2+}. a, Average normalized currents showing rundown behaviors of TMEM16A WT and mutations of the putative PIP_2 binding residues in the presence of 0.5 μM Ca^{2+}. **b**, Quantifications of the residual currents following 5 minutes of channel activation. One-way ANOVA with Bonferroni's multiple comparisons test: p-values are <0.0001 for R451A, <0.0001 for K461A, <0.0001 for R482A, 0.0003 for K567A, 0.0008 for R575A, and <0.0001 for K579A. Numbers in parentheses denote the number of individual recordings. Data are mean ± s.e.m. Source data are provided as a Source Data file.

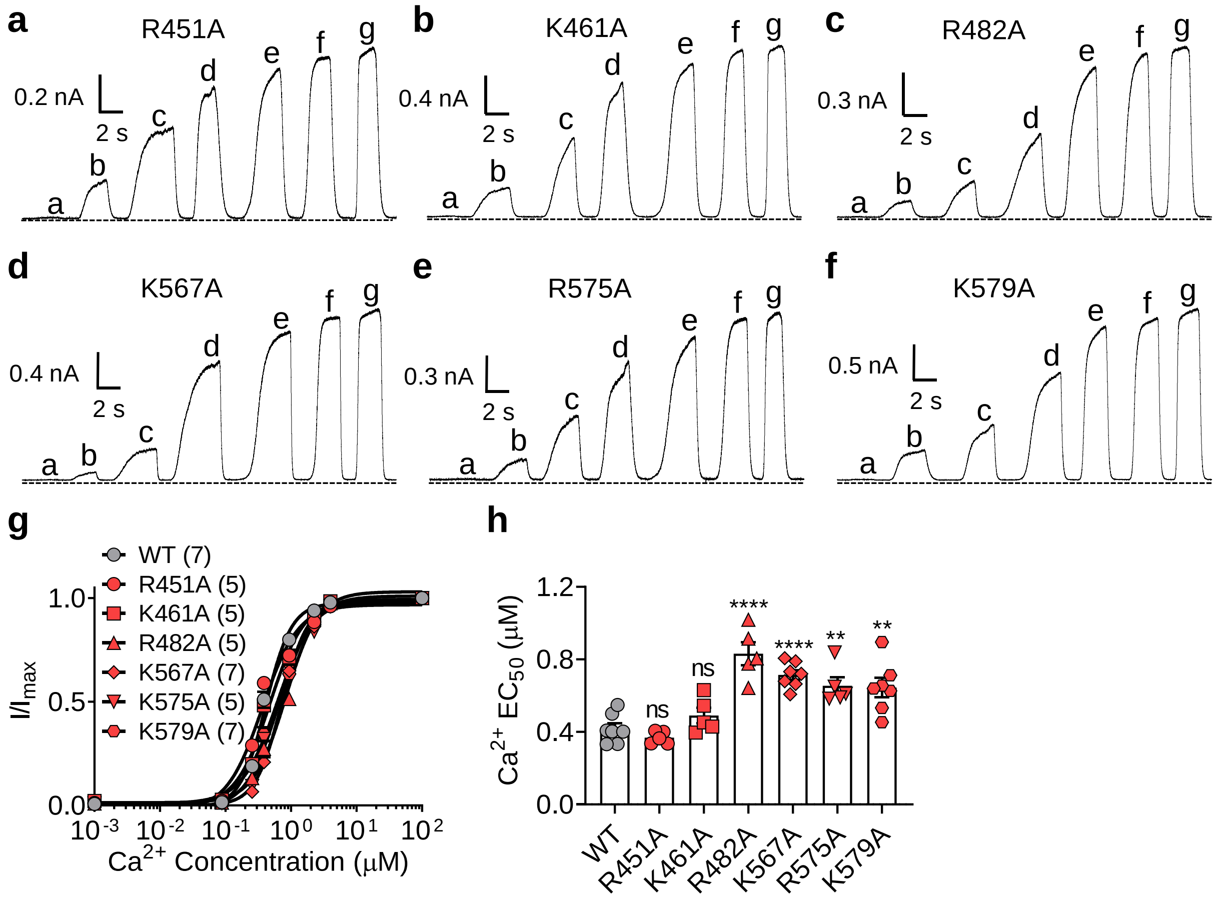

Supplementary Fig. 8. The apparent Ca^{2+} sensitivities of the putative PIP_2 binding mutants. **a**-**f**, Representative Ca^{2+} dose-response recordings of R451A (**a**), K461A (**b**), R482A (**c**), K567A (**d**), R575A (**e**), and K579A (**f**). Membrane was held at +60 mV, and solutions with various Ca^{2+} concentrations were sequentially applied to trigger TMEM16A activation. Ca^{2+}-free solution was perfused to deactivate the channels after each activation. Letters (a to g) above each upward deflection currents denote free Ca^{2+} concentrations of 0.088, 0.0255, 0.387, 0.94, 2.26, 4 and 100 μM, respectively. **g**,**h**, Ca^{2+} dose-responses (**g**) of WT and TMEM16A mutants and their half maximal effective concentrations of Ca^{2+} (Ca^{2+} EC_{50}) of channel activation (**h**). The smooth curves represent fits to the Hill equation. One-way ANOVA with Bonferroni's multiple comparisons test: p-values are >0.9999 for R451A, >0.9999 for K461A, <0.0001 for R482A, <0.0001 for K567A, 0.0022 for R575A, and 0.0012 for K579A. Numbers in parentheses denote the number of individual recordings. Data are mean ± s.e.m. ns, not significant. Source data are provided as a Source Data file.

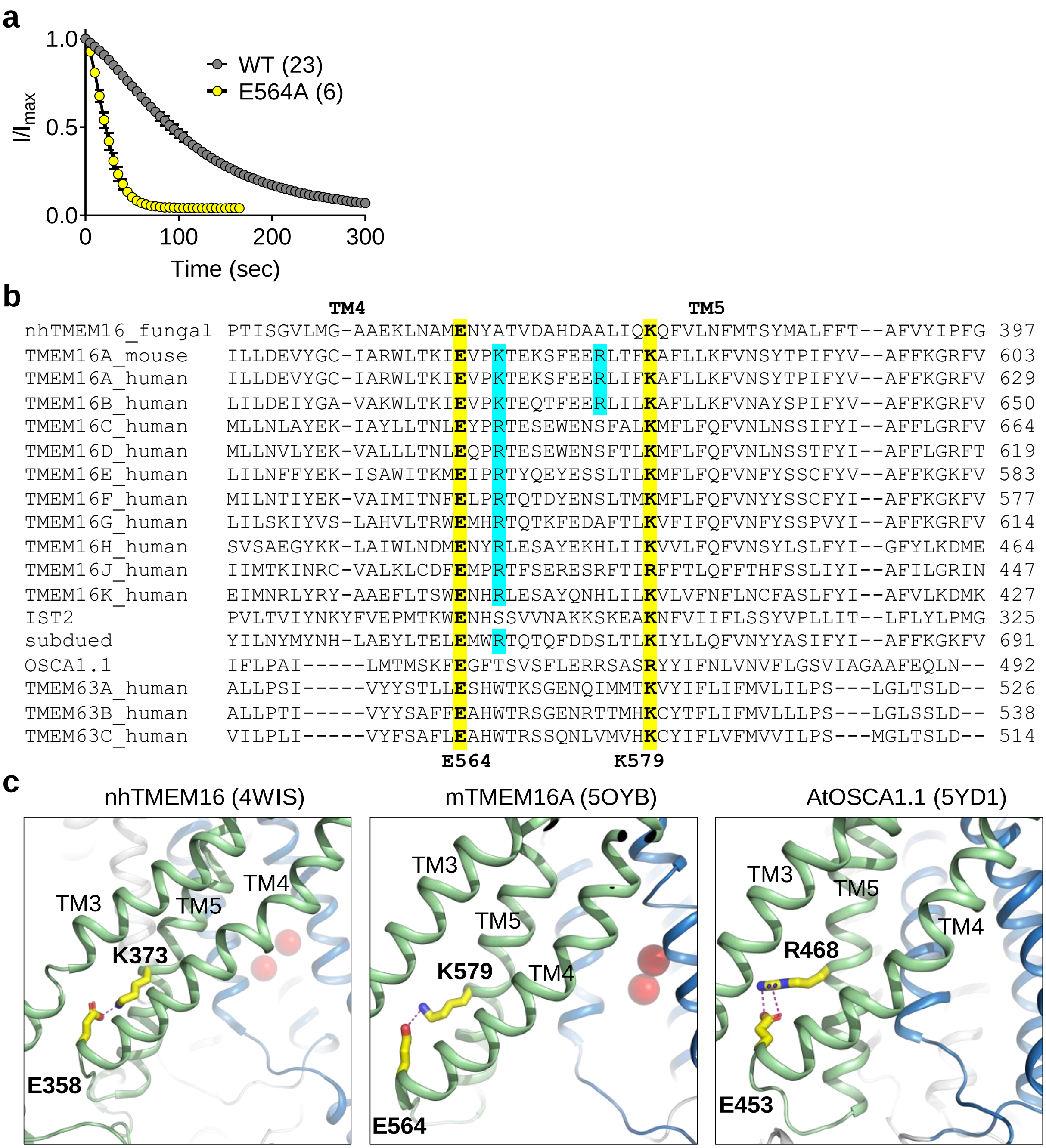

Supplementary Fig. 9. An evolutionarily conserved salt bridge between TM4 and TM5. a, Alanine mutation of E564 in TMEM16A greatly accelerated desensitization. Number in parentheses denote the number of individual recordings. Data are mean ± s.e.m. **b**, Protein sequence alignment of TMEM16 proteins, including the fungal scramblase nhTMEM16, all mammalian TMEM16 proteins, the yeast TMEM16 homolog IST2, drosophila TMEM16 homolog subdued, the plant osmo/mechanosensitive ion channel OSCA1.1, and the TMEM16-related mechanosensitive ion channels TMEM63A to B. Highlighted in yellow are the highly conserved salt bridge residues (E564 and K579 in mTMEM16A) from TM4 and TM5, respectively. Highlighted in cyan are K567 and R575 (in mTMEM16A), two of the proposed putative PIP_2 binding residues. **c**, Structures of the fungal *Nectria haematococca* TMEM16 (nhTMEM16, PDB

code 4WIS), mouse TMEM16A (mTMEM16A, PDB code 5OYB), and *Arabidopsis thaliana* OSCA1.1 (AtOSCA1.1, PDB code 5YD1) showing architectural similarities and a highly conserved salt bridge (yellow sticks) between TM4 and TM5: E358 and K373 in nhTMEM16, E564 and K579 in mTMEM16A, and E453 and R468 in AtOSCA1.1.

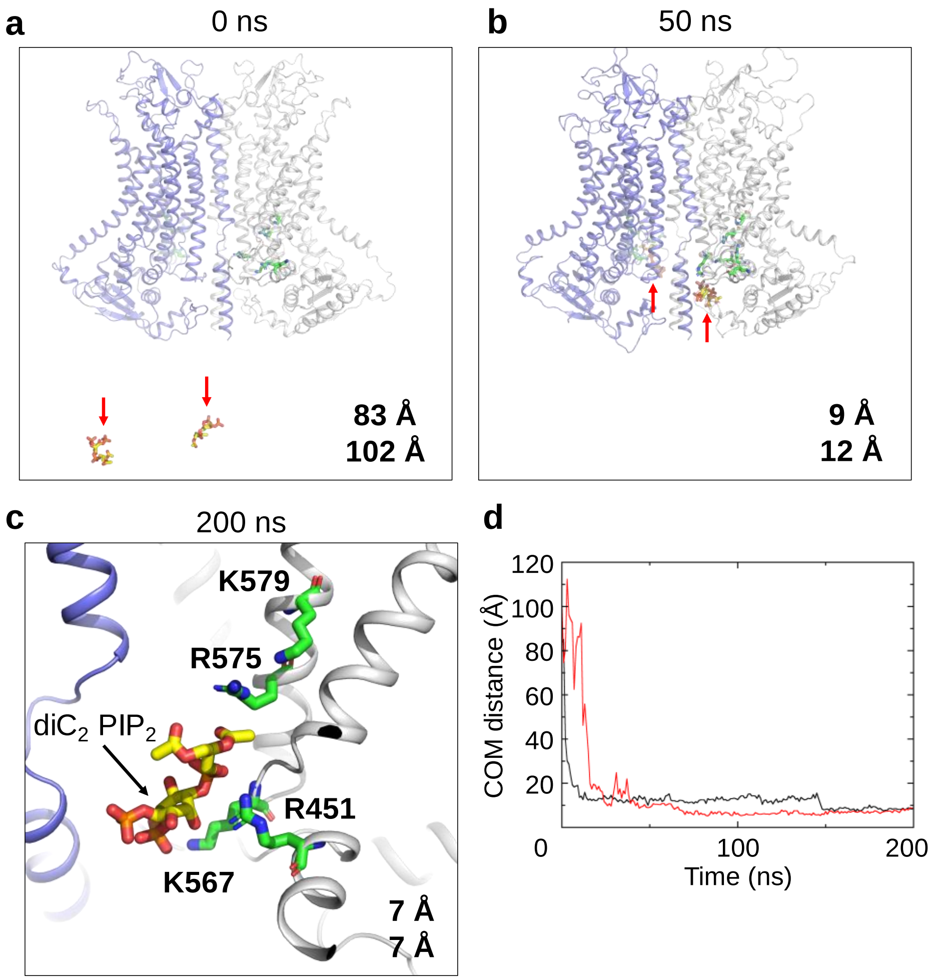

Supplementary Fig. 10. Spontaneous binding of two PIP_2 headgroups to the putative PIP_2 binding sites. a-c, Snapshots taken at 0 (**a**), 50 (**b**) and 200 ns (**c**) of simulation showing the two spontaneous PIP_2 binding events. The distances between the center of mass (COM) of PIP_2 head groups (diC_2 PIP_2) and key PIP_2 binding residues are shown at the bottom right of each panel. Red arrows indicate positions of the two PIP_2 head groups. The lipid bilayer was excluded from visualization. The shortest possible distances between PIP_2 headgroup and R451, R482, K567, R575, and K579 are 4.1 ± 1.9, 8.7 ± 6.2, 2.8 ± 0.6, 2.7 ± 0.2, 6.6 ± 1.1 Å. **d**, Distance between the COM of PIP_2 head group and key PIP_2 binding residues as a function of time for two spontaneous binding events observed during unrestrained atomistic simulations. Both PIP_2 head groups diffused rapidly from the bulk solution and entered PIP_2 binding pockets in TMEM16A dimer to form direct contacts with the putative PIP_2 binding residues.

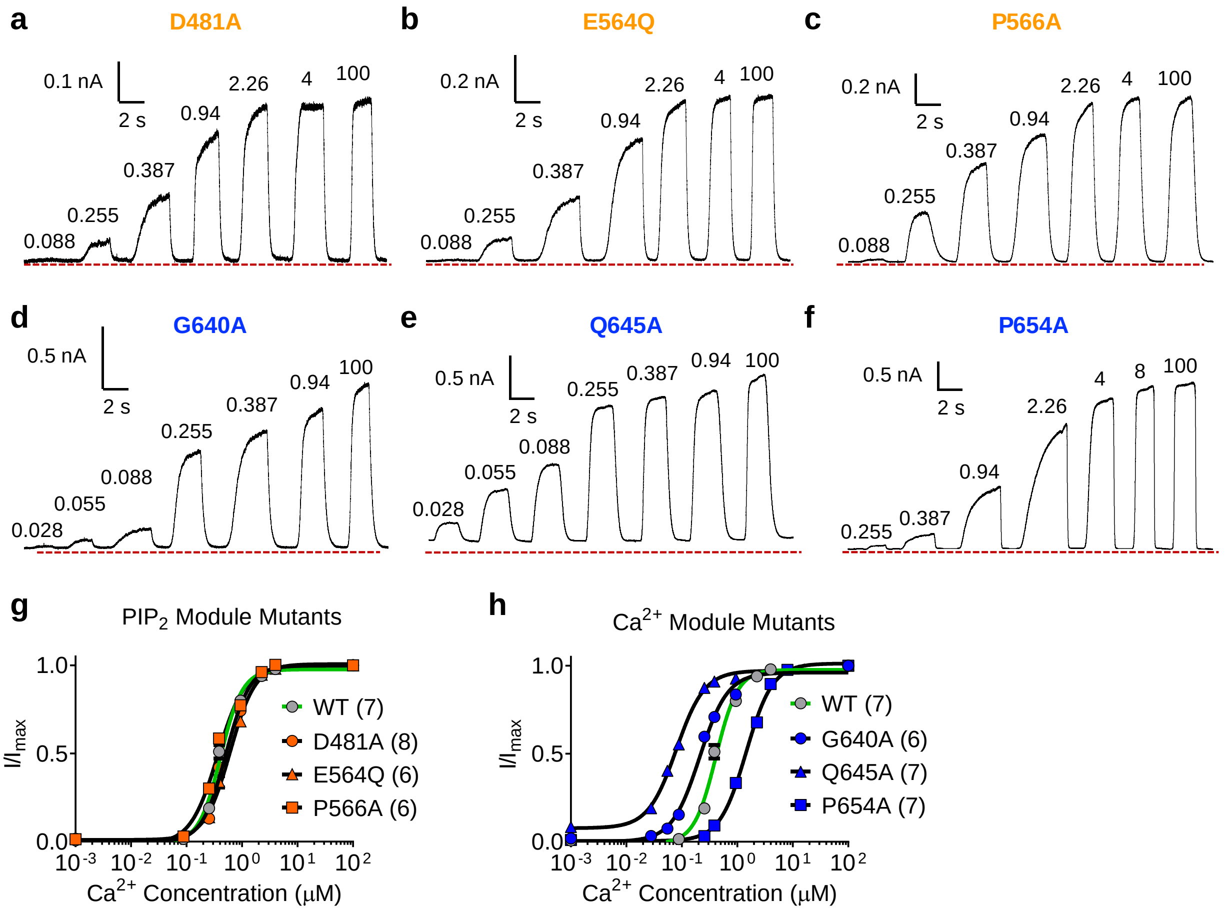

Supplementary Fig. 11. Mutations of the Ca^{2+} module, but not the PIP_2 module, alter the TMEM16A's Ca^{2+} sensitivity. a-f, Representative Ca^{2+} dose-response recordings of D481A (**a**), E564Q (**b**), P566A (**c**), G640A (**d**), Q645A (**e**), and P654A (**f**). Membrane was held at +60 mV, and solutions with various Ca^{2+} concentrations (numbers on top, in µM) were sequentially applied to trigger TMEM16A activation. Ca^{2+}-free solution was perfused to close the channels after each activation. Note the different ranges of Ca^{2+} concentrations used for G640A, Q645A, and P654A due to their altered Ca^{2+} sensitivities. **g**,**h**, Ca^{2+} dose-responses of the mutations from the PIP_2 module including D481A, E564Q, and P566A (red data points, **g**) and the mutations from the Ca^{2+} module including G640A, Q645A, and P654A (blue data points, **h**) in comparison to WT (green curve with gray data points). The sigmoidal curves represent fits to the Hill equation. Data are mean ± s.e.m. Source data are provided as a Source Data file.

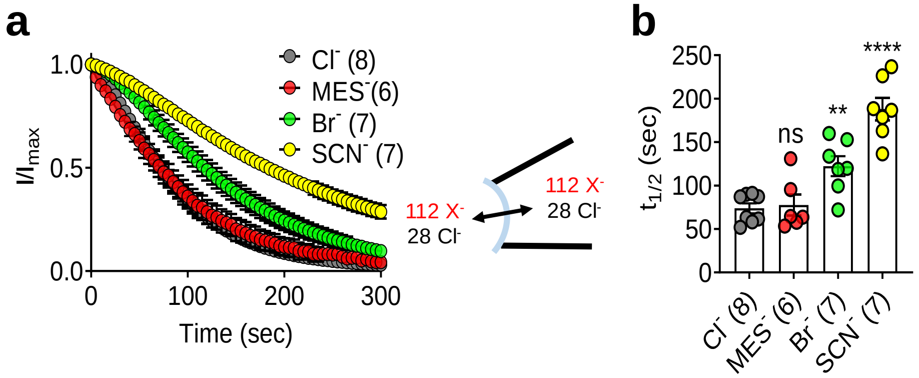

Supplementary Fig. 12. Effects of large anions on TMEM16A rundown. a, Average normalized currents showing the effects of the large and more permeable anions SCN^- and Br^- and the impermeant MES^- on TMEM16A desensitization. Inset shows the ionic scheme of symmetric solutions containing 112 mM of X^- (SCN^-, Br^-, or MES^-) in addition to 28 mM Cl^-. Perfusion solution (intracellular) contained 100 μM Ca^{2+} to elicit channel activation. Numbers in parentheses denote the number of individual recordings. **b**, Quantifications of the half-decay time ($t_{1/2}$) of recordings in **a**. One-way ANOVA with Bonferroni's multiple comparisons test: p-values are >0.9999 for MES^-, 0.0085 for Br^-, and <0.0001 for SCN^-. Data are mean ± s.e.m. ns, not significant. Source data are provided as a Source Data file.

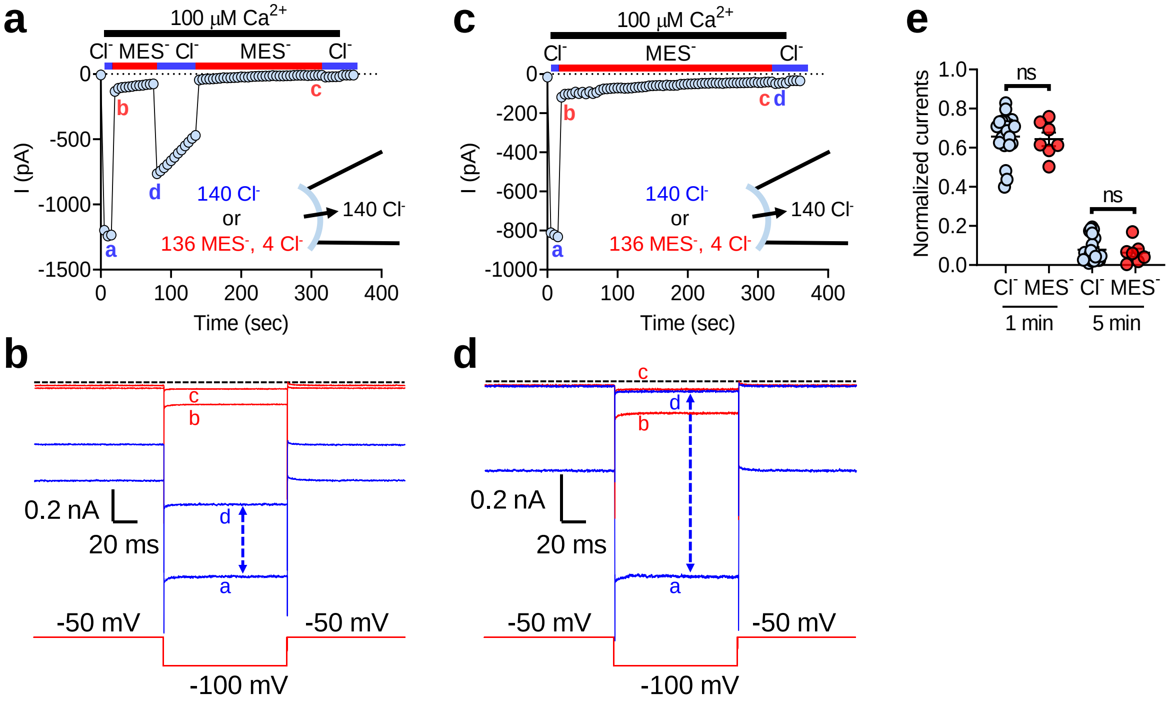

Supplementary Fig. 13. Reduced ion permeation does not affect TMEM16A rundown. a-d, Representative recordings showing time-course of TMEM16A channel activity when intracellular Cl^- was replaced with MES^--containing solution for 1 minute (**a** and **b**) or for 5 minutes (**c** and **d**), followed by reintroduction of Cl^- to estimate the remaining channel activity. Representative current traces at each time point in **a** and **b** are shown in **c** and **d**. Insets show the schematic ionic contents of the pipette solution (140 mM Cl^-) and the perfusion solutions containing either 140 mM Cl^- or 136 MES^- and 4 mM Cl^-. Black arrows indicate direction of Cl^- movement when the modified voltage step protocol was used. Perfusion solutions also contained 100 μM Ca^{2+} to elicit channel opening. Due to presence of 140 mM Cl^- in the external side (pipette solution) and to avoid Cl^- influx (from pipette to the intracellular side), a modified voltage step protocol was utilized in which the membrane was held at -50 mV and a voltage step of -100 mV was applied at an interval of 5 seconds. Peak inward currents (representing Cl^- efflux) measured at -100 mV were used for time-course monitoring and quantifications. **e**, Quantifications of current inhibition ratios following 1 minute or 5 minutes of MES^- perfusion. Data points for Cl^- control condition are extracted from WT recordings in symmetric 140 mM Cl^- shown in **Fig. 1f**. Two-tailed unpaired Student's *t*-test: p-values are 0.7246 for 1 min and 0.4477 for 5 min. ns, not significant. Source data are provided as a Source Data file.

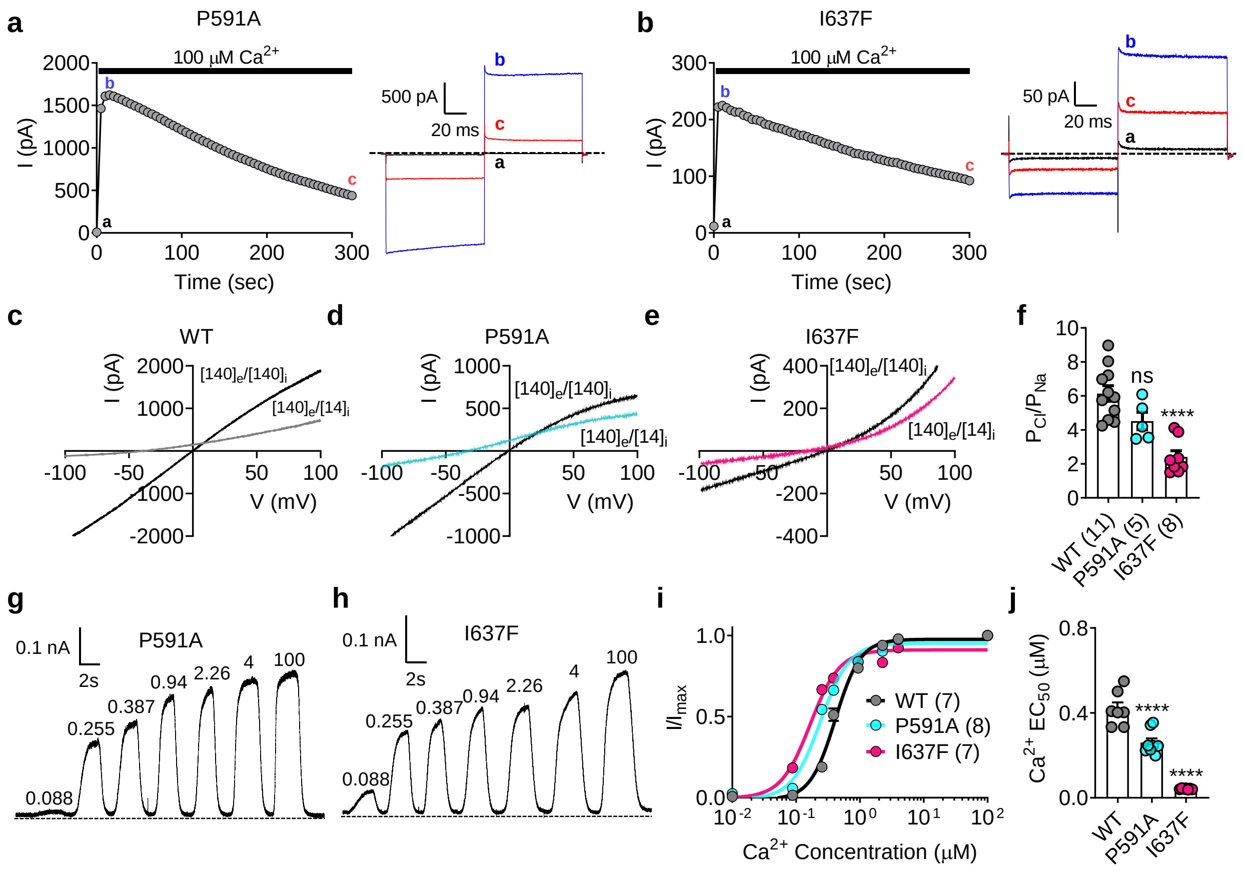

Supplementary Fig. 14. Functional characterizations of the pore residues P591 and I637. a,**b**, Representative recordings of P591A (**a**) and I637F (**b**) mutations under saturating 100 μM Ca^{2+}. Current traces at different time points are shown as insets on the right. **c**-**e**, Measurements of the shifts in the reversal potential (E_{rev}) of TMEM16A WT (**c**), P591A (**d**), I637F (**e**) when switching the solutions from symmetric 140 mM NaCl (both intracellular and extracellular) to asymmetric 14 mM NaCl (intracellular)/140 mM (extracellular). **f**, Quantifications of permeability ratios (P_{Cl}/P_{Na}) of TMEM16A WT, P591A, and I637F calculated from the shifts in their E_{rev} (ΔE_{rev}) using the Goldman–Hodgkin–Katz (GHK) equation. One-way ANOVA with Bonferroni's multiple comparisons test: p-values are 0.0638 and <0.0001 for P591A and I637F, respectively. **g-j**, Measurements of the Ca^{2+} sensitivities of TMEM16A P591A and I637F mutations. Membrane was held at +60 mV, and solutions with various Ca^{2+} concentrations (numbers on top, in μM) were sequentially applied to trigger TMEM16A activation (**g** and **h**). Ca^{2+}-free solution was perfused to close the channels after each activation. The sigmoidal curves represent fits to the Hill equation (**i**). One-way ANOVA with Bonferroni's multiple comparisons test: p-values are both <0.0001 for both P591A and I637F. Data are mean ± s.e.m. ns, not significant. Source data are provided as a Source Data file.

Supplementary Table 1. Primers for QuikChange mutagenesis

	Mutations	Primers
1	R298A	Forward: 5' CGTTGAGTTCAACGACGCGAAACTCCTGTATGAG 3'
		Reverse: 5' CTCATACAGGAGTTTCGCGTCGTTGAACTCAACG 3'
2	K299A	Forward: 5' GAGTTCAACGACAGGGCACTCCTGTATGAGG 3'
		Reverse: 5' CCTCATACAGGAGTGCCCTGTCGTTGAACTC 3'
3	K313A	Forward: 5' GTTACGGAGTCTTCTACGCATACCAGCCCATTGAC 3'
		Reverse: 5' GTCAATGGGCTGGTATGCGTAGAAGACTCCGTAAC 3'
4	R321A	Forward: 5' CCCATTGACCTGGTCGCGAAATACTTTGGTG 3'
		Reverse: 5' CACCAAAGTATTTCGCGACCAGGTCAATGGG 3'
5	K322A	Forward: 5' CATTGACCTGGTCAGGGCATACTTTGGTGAGAAG 3'
		Reverse: 5' CTTCTCACCAAAGTATGCCCTGACCAGGTCAATG 3'
6	K327A	Forward: 5' GAAATACTTTGGTGAGGCGGTTGGCCTGTACTTTG 3'
		Reverse: 5' CAAAGTACAGGCCAACCGCCTCACCAAAGTATTTC 3'
7	K428A	Forward: 5' CTTTCATGGAGCACTGGGCACGGAAGCAGATGAGG 3'
		Reverse: 5' CCTCATCTGCTTCCGTGCCCAGTGCTCCATGAAAG 3'
8	K429A	Forward: 5' GGAGCACTGGAAAGCGAAGCAGATGAGGC 3'
		Reverse: 5' GCCTCATCTGCTTCGCTTTCCAGTGCTCC 3'
9	K430L	Forward: 5' GAGCACTGGAAACGGCTGCAGATGAGGCTCAAC 3'
		Reverse: 5' GTTGAGCCTCATCTGCAGCCGTTTCCAGTGCTC 3'
10	R433A	Forward: 5' GAAACGGAAGCAGATGGCGCTCAACTACCGATG 3'
		Reverse: 5' CATCGGTAGTTGAGCGCCATCTGCTTCCGTTTC 3'
11	R437A	Forward: 5' GAGGCTCAACTACGCATGGGACCTCACAG 3'
		Reverse: 5' CTGTGAGGTCCCATGCGTAGTTGAGCCTC 3'
12	R451A	Forward: 5' GAGGAGGATCATCCCGCAGCAGAGTATGAAG 3'
		Reverse: 5' CTTCATACTCTGCTGCGGGATGATCCTCCTC 3'
13	R457A	Forward: 5' GAGCAGAGTATGAAGCCGCAGTCTTAGAGAAGTCAC 3'
		Reverse: 5' GTGACTTCTCTAAGACTGCGGCTTCATACTCTGCTC 3'
14	K461A	Forward: 5' GCCAGAGTCTTAGAGGCGTCACTGAGAAAAG 3'
		Reverse: 5' CTTTTCTCAGTGACGCCTCTAAGACTCTGGC 3'
15	R464A	Forward: 5' GAGTCTTAGAGAAGTCACTGGCAAAAGAATCCAGAAACAAAG 3'
		Reverse: 5' CTTTGTTTCTGGATTCTTTTGCCAGTGACTTCTCTAAGACTC 3'
16	K465A	Forward: 5' CTTAGAGAAGTCACTGAGAGCAGAATCCAGAAACAAAGAG 3'
		Reverse: 5' CTCTTTGTTTCTGGATTCTGCTCTCAGTGACTTCTCTAAG 3'
17	R468A	Forward: 5' CACTGAGAAAAGAATCCGCAAACAAAGAGACCGAC 3'
		Reverse: 5' GTCGGTCTCTTTGTTTGCGGATTCTTTTCTCAGTG 3'
18	K470A	Forward: 5' GAAAAGAATCCAGAAACGCAGAGACCGACAAGGTG 3'
		Reverse: 5' CACCTTGTCGGTCTCTGCGTTTCTGGATTCTTTTC 3'
19	K474A	Forward: 5' GAGCAGAGTATGAAGCCGCAGTCTTAGAGAAGTCAC 3'

		Reverse: 5' GTGACTTCTCTAAGACTGCGGCTTCATACTCTGCTC 3'
20	K476A	Forward: 5' CAAAGAGACCGACAAGGTGGCGCTGACCTGGAGGGACCG 3'
21		Reverse: 5' CGGTCCCTCCAGGTCAGCGCCACCTTGTCGGTCTCTTTG 3'
	R480A	Forward: 5' GTGAAGCTGACCTGGGCGGACCGATTCCCAG 3'
22		Reverse: 5' CTGGGAATCGGTCCGCCCAGGTCAGCTTCAC 3'
	R482A	Forward: 5' CTGACCTGGAGGGACGCATTCCCAGCCTATTTC 3'
23		Reverse: 5' GAAATAGGCTGGGAATGCGTCCCTCCAGGTCAG 3'
	R558A	Forward: 5' GTTTACGGCTGCATTGCCGCGTGGCTCACCAAGATTG 3'
24		Reverse: 5' CAATCTTGGTGAGCCACGCGGCAATGCAGCCGTAAAC 3'
	K562A	Forward: 5' CATTGCCAGGTGGCTCACCGCGATTGAGGTCCCAAAGAC 3'
25		Reverse: 5' GTCTTTGGGACCTCAATCGCGGTGAGCCACCTGGCAATG 3'
	K567A	Forward: 5' CAAGATTGAGGTCCCAGCGACAGAGAAGAGCTTTG 3'
26		Reverse: 5' CAAAGCTCTTCTCTGTCGCTGGGACCTCAATCTTG 3'
	K570A	Forward: 5' GTCCCAAAGACAGAGGCGAGCTTTGAGGAGAG 3'
27		Reverse: 5' CTCTCCTCAAAGCTCGCCTCTGTCTTTGGGAC 3'
	R575A	Forward: 5' GAAGAGCTTTGAGGAGGCGCTAACCTTCAAGGC 3'
28		Reverse: 5' GCCTTGAAGGTTAGCGCCTCCTCAAAGCTCTTC 3'
	K579A	Forward: 5' GAGGCTAACCTTCGCGGCCTTCCTGCTC 3'
29		Reverse: 5' GAGCAGGAAGGCCGCGAAGGTTAGCCTC 3'
	K584A	Forward: 5' CAAGGCCTTCCTGCTCGCGTTTGTGAACTCTTAC 3'
30		Reverse: 5' GTAAGAGTTCACAAACGCGAGCAGGAAGGCCTTG 3'
	K655A	Forward: 5' CGAGATTGGCATCCCGGCGATGAAAAAGTTCATC 3'
31		Reverse: 5' GATGAACTTTTTCATCGCCGGGATGCCAATCTCG 3'
	K657A	Forward: 5' GGCATCCCGAAGATGGCAAAGTTCATCCGCTAC 3'
		Reverse: 5' GTAGCGGATGAACTTTGCCATCTTCGGGATGCC 3'
32	K658A	Forward: 5' CATCCCGAAGATGAAAGCGTTCATCCGCTACCTG 3'
		Reverse: 5' CAGGTAGCGGATGAACGCTTTCATCTTCGGGATG 3'
33	R661A	Forward: 5' GATGAAAAAGTTCATCGCCTACCTGAAGCTGCGC 3'
		Reverse: 5' GCGCAGCTTCAGGTAGGCGATGAACTTTTTCATC 3'
34	K664A	Forward: 5' GAAAAAGTTCATCCGCTACCTGGCGCTGCGCAGACAGAGCCCCTC 3'
		Reverse: 5' GAGGGGCTCTGTCTGCGCAGCGCCAGGTAGCGGATGAACTTTTTC 3'
35	R666A	Forward: 5' CTACCTGAAGCTGGCCAGACAGAGCCC 3'
		Reverse: 5' GGGCTCTGTCTGGCCAGCTTCAGGTAG 3'
36	R667A	Forward: 5' CTACCTGAAGCTGCGCGCACAGAGCCCCTCAGAC 3'
		Reverse: 5' GTCTGAGGGGCTCTGTGCGCGCAGCTTCAGGTAG 3'
37	R673A	Forward: 5' CAGAGCCCCTCAGACGCTGAAGAGTACGTGAAG 3'
		Reverse: 5' CTTCACGTACTCTTCAGCGTCTGAGGGGCTCTG 3'
38	K678A	Forward: 5' CCGTGAAGAGTACGTGGCGCGGAAGCAGCGCTATG 3'
		Reverse: 5' CATAGCGCTGCTTCCGCGCCACGTACTCTTCACGG 3'

39	R679A	Forward: 5' GAAGAGTACGTGAAGGCGAAGCAGCGCTATG 3'
		Reverse: 5' CATAGCGCTGCTTCGCCTTCACGTACTCTTC 3'
40	K680A	Forward: 5' GAGTACGTGAAGCGGGCGCAGCGCTATGAGGTG 3'
		Reverse: 5' CACCTCATAGCGCTGCGCCCGCTTCACGTACTC 3'
41	R682A	Forward: 5' GTGAAGCGGAAGCAGGCCTATGAGGTGGACTTC 3'
		Reverse: 5' GAAGTCCACCTCATAGGCCTGCTTCCGCTTCAC 3'
42	R732A	Forward: 5' CTAAACAACATCATTGAGATCGCCCTGGATGCCAAAAAGTTTG 3'
		Reverse: 5' CAAACTTTTTGGCATCCAGGGCGATCTCAATGATGTTGTTTAG 3'
43	K736A	Forward: 5' GATCCGCCTGGATGCCGCAAAGTTTGTCACCGAG 3'
		Reverse: 5' CTCGGTGACAAACTTTGCGGCATCCAGGCGGATC 3'
44	K737A	Forward: 5' CCTGGATGCCAAAGCGTTTGTCACCGAGC 3'
		Reverse: 5' GCTCGGTGACAAACGCTTTGGCATCCAGG 3'
45	R743A	Forward: 5' GTTTGTCACCGAGCTAGCGAGGCCAGTAGCCATC 3'
		Reverse: 5' GATGGCTACTGGCCTCGCTAGCTCGGTGACAAAC 3'
46	R744A	Forward: 5' GTTTGTCACCGAGCTACGGGCGCCAGTAGCCATCAGAGC 3'
		Reverse: 5' GCTCTGATGGCTACTGGCGCCCGTAGCTCGGTGACAAAC 3'
47	R749A	Forward: 5' GCCAGTAGCCATCGCAGCCAAAGACATCG 3'
		Reverse: 5' CGATGTCTTTGGCTGCGATGGCTACTGGC 3'
48	K751A	Forward: 5' GTAGCCATCAGAGCCGCAGACATCGGCATCTG 3'
		Reverse: 5' CAGATGCCGATGTCTGCGGCTCTGATGGCTAC 3'
49	R761A	Forward: 5' CTGGTATAACATCCTCGCAGGTGTTGGGAAGCTG 3'
		Reverse: 5' CAGCTTCCCAACACCTGCGAGGATGTTATACCAG 3'
50	R765A	Forward: 5' CATCCTCAGAGGTGTTGGGGCGCTGGCTGTCATCATTAATG 3'
		Reverse: 5' CATTAATGATGACAGCCAGCGCCCCAACACCTCTGAGGATG 3'
51	K887A	Forward: 5' GATCCCTGATATCCCCGCAGACATCAGCCAGCAG 3'
		Reverse: 5' CTGCTGGCTGATGTCTGCGGGGATATCAGGGATC 3'
52	K895A	Forward: 5' GCCAGCAGATCCACGCAGAGAAGGTTCTC 3'
		Reverse: 5' GAGAACCTTCTCTGCGTGGATCTGCTGGC 3'
53	D481A	Forward: 5' CTGACCTGGAGGGCCCGATTCCCAGC 3'
		Reverse: 5' GCTGGGAATCGGGCCCTCCAGGTCAG 3'
54	E564A	Forward: 5' CTCACCAAGATTGCGGTCCCAAAGACAG 3'
		Reverse: 5' CTGTCTTTGGGACCGCAATCTTGGTGAG 3'
55	E564Q	Forward: 5' GGCTCACCAAGATTCAGGTCCCAAAGAC 3'
		Reverse: 5' GTCTTTGGGACCTGAATCTTGGTGAGCC 3'
56	P566A	Forward: 5' CACCAAGATTGAGGTCGCAAAGACAGAGAAGAG 3'
		Reverse: 5' CTCTTCTCTGTCTTTGCGACCTCAATCTTGGTG 3'
57	G640A	Forward: 5' GCATCATTATGCTGGCCAAGCAGCTAATCC 3'
		Reverse: 5' GGATTAGCTGCTTGGCCAGCATAATGATGC 3'

Q645A	Forward: 5' GGCAAGCAGCTAATCGCGAACAATCTCTTCG 3'
	Reverse: 5' CGAAGAGATTGTTCGCGATTAGCTGCTTGCC 3'
P591A	Forward: 5' GTGAACTCTTACACTGCCATCTTCTATGTCG 3'
	Reverse: 5' CGACATAGAAGATGGCAGTGTAAGAGTTCAC 3'
I637F	Forward: 5' CCAGCTGAGCATCTTTATGCTGGGCAAG 3'
	Reverse: 5' CTTGCCCAGCATAAAGATGCTCAGCTGG 3'
