# Supplementary material for: Molecular basis of PIP2-dependent regulation of the Ca2+-activated chloride channel TMEM16A
Source: Nat Commun. 2019 Aug 21;10:3769. doi: 10.1038/s41467-019-11784-8 (PMC6704070; doi:10.1038/s41467-019-11784-8)
Supplement: Supplementary file 4 — Reporting Summary [file 41467_2019_11784_MOESM4_ESM.pdf]

nature research

Corresponding author(s): Huanghe Yang

Last updated by author(s): Jul 26, 2019

Reporting Summary

Nature Research wishes to improve the reproducibility of the work that we publish. This form provides structure for consistency and transparency in reporting. For further information on Nature Research policies, see Authors & Referees and the Editorial Policy Checklist.

Statistics

For all statistical analyses, confirm that the following items are present in the figure legend, table legend, main text, or Methods section.

n/a	Confirmed	
☐	☒	The exact sample size (*n*) for each experimental group/condition, given as a discrete number and unit of measurement
☐	☒	A statement on whether measurements were taken from distinct samples or whether the same sample was measured repeatedly
☐	☒	The statistical test(s) used AND whether they are one- or two-sided<br>*Only common tests should be described solely by name; describe more complex techniques in the Methods section.*
☒	☐	A description of all covariates tested
☒	☐	A description of any assumptions or corrections, such as tests of normality and adjustment for multiple comparisons
☐	☒	A full description of the statistical parameters including central tendency (e.g. means) or other basic estimates (e.g. regression coefficient) AND variation (e.g. standard deviation) or associated estimates of uncertainty (e.g. confidence intervals)
☐	☒	For null hypothesis testing, the test statistic (e.g. *F*, *t*, *r*) with confidence intervals, effect sizes, degrees of freedom and *P* value noted<br>*Give P values as exact values whenever suitable.*
☒	☐	For Bayesian analysis, information on the choice of priors and Markov chain Monte Carlo settings
☒	☐	For hierarchical and complex designs, identification of the appropriate level for tests and full reporting of outcomes
☒	☐	Estimates of effect sizes (e.g. Cohen's *d*, Pearson's *r*), indicating how they were calculated

Our web collection on statistics for biologists contains articles on many of the points above.

Software and code

Policy information about availability of computer code

Data collection	Axon PClamp 10 (Molecular Devices)
Data analysis	Axon Clampfit 10 (Molecular Devices); MATLAB R2018b (Mathworks); Prism 8 (Graphpad); Excel (Microsoft); Pymol (Schrodinger); AutoDock Vina; CHARM-GUI

For manuscripts utilizing custom algorithms or software that are central to the research but not yet described in published literature, software must be made available to editors/reviewers. We strongly encourage code deposition in a community repository (e.g. GitHub). See the Nature Research guidelines for submitting code & software for further information.

Data

Policy information about availability of data

All manuscripts must include a data availability statement. This statement should provide the following information, where applicable:
- Accession codes, unique identifiers, or web links for publicly available datasets
- A list of figures that have associated raw data
- A description of any restrictions on data availability

All relevant data collected, analyzed and shown in main and supplementary figures as well as code used for analysis are available from the corresponding author upon reasonable request.

Field-specific reporting

Please select the one below that is the best fit for your research. If you are not sure, read the appropriate sections before making your selection.

☒ Life sciences ☐ Behavioural & social sciences ☐ Ecological, evolutionary & environmental sciences

For a reference copy of the document with all sections, see nature.com/documents/nr-reporting-summary-flat.pdf

Life sciences study design

All studies must disclose on these points even when the disclosure is negative.

Sample size	No statistical methods were used to predetermine sample size. The number of recordings of each experiment was based on personal experience and data variability. Each experiment was performed on at least 4 to 23 individual cells.
Data exclusions	Electrophysiology recordings displaying high noises, instability, or current amplitudes too small for analyses were excluded.
Replication	All experiments were successfully reproduced with similar results.
Randomization	The experiments were not randomized.
Blinding	The investigators were not blinded to allocation during experiments and outcome assessment.

Reporting for specific materials, systems and methods

We require information from authors about some types of materials, experimental systems and methods used in many studies. Here, indicate whether each material, system or method listed is relevant to your study. If you are not sure if a list item applies to your research, read the appropriate section before selecting a response.

Materials & experimental systems

n/a	Involved in the study	
☒	☐	Antibodies
☐	☒	Eukaryotic cell lines
☒	☐	Palaeontology
☒	☐	Animals and other organisms
☒	☐	Human research participants
☒	☐	Clinical data

Methods

n/a	Involved in the study	
☒	☐	ChIP-seq
☒	☐	Flow cytometry
☒	☐	MRI-based neuroimaging

Eukaryotic cell lines

Policy information about cell lines

Cell line source(s)	HEK293T cells obtained from the Duke Cell Culture Facility (CCF).
Authentication	Cells were authenticated by Duke CCF; no further authentication was performed.
Mycoplasma contamination	Cells were tested negative for mycoplasma by Duke CCF and no further tests for mycoplasma were performed.
Commonly misidentified lines (See ICLAC register)	N/A
