# Supplementary material for: Molecular basis of PIP2-dependent regulation of the Ca2+-activated chloride channel TMEM16A
Source: Nat Commun. 2019 Aug 21;10:3769. doi: 10.1038/s41467-019-11784-8 (PMC6704070; doi:10.1038/s41467-019-11784-8)
Supplement: Supplementary file 5 — Description of Additional Supplementary Files [file 41467_2019_11784_MOESM5_ESM.pdf]

Title: Supplementary Movie 1.
Description: Spontaneous binding of a full-length PIP_2 molecule when placed in proximity of the putative PIP_2 binding site. Putative PIP_2 binding residues R451, K567, and R575 are shown as sticks. See Fig. 4 for more details.

Title: Supplementary Movie 2.
Description: Binding of PIP_2 head-groups (diC_2 PIP_2) when placed randomly in the bulk solvent. Rapid diffusion of two PIP_2 head-groups within 200 ns of simulation to occupy the putative PIP_2 binding sites. Putative PIP_2 binding residues are shown in sticks. See Supplementary Fig. 10 for more details.
